# Subclonal heterogeneity and evolution in breast cancer

**DOI:** 10.1038/s41523-021-00363-0

**Published:** 2021-12-21

**Authors:** Ioanna Mavrommati, Flora Johnson, Gloria V. Echeverria, Rachael Natrajan

**Affiliations:** 1 The Breast Cancer Now Toby Robins Research Centre, The Institute of Cancer Research, London, UK; 2 Lester and Sue Smith Breast Center, Baylor College of Medicine, Houston, TX USA; 3 Department of Medicine, Baylor College of Medicine, Houston, TX USA; 4 Dan L. Duncan Cancer Center, Baylor College of Medicine, Houston, TX USA; 5 Department of Molecular and Cellular Biology, Baylor College of Medicine, Houston, TX USA

**Keywords:** Breast cancer, Tumour heterogeneity

## Abstract

Subclonal heterogeneity and evolution are characteristics of breast cancer that play a fundamental role in tumour development, progression and resistance to current therapies. In this review, we focus on the recent advances in understanding the epigenetic and transcriptomic changes that occur within breast cancer and their importance in terms of cancer development, progression and therapy resistance with a particular focus on alterations at the single-cell level. Furthermore, we highlight the utility of using single-cell tracing and molecular barcoding methodologies in preclinical models to assess disease evolution and response to therapy. We discuss how the integration of single-cell profiling from patient samples can be used in conjunction with results from preclinical models to untangle the complexities of this disease and identify biomarkers of disease progression, including measures of intra-tumour heterogeneity themselves, and how enhancing this understanding has the potential to uncover new targetable vulnerabilities in breast cancer.

## Introduction

Breast cancer is a heterogeneous disease, driven by a myriad of genetic and non-genetic alterations that govern its clinical behaviour^1^. Tumour heterogeneity (TH) can exist at different levels (for example; genomic, transcriptomic and proteomic) and refers to the observation that cancer cells can have distinct molecular profiles from one another. This diversity of tumour cells’ profiles, can be either observed between tumours, known as inter-tumour heterogeneity (inter-TH), or within the same tumours, known as intra-tumour heterogeneity (intra-TH)^2^.

Inter-TH in breast cancer has led to the classification based on histology and expression profiles of the molecular markers; oestrogen receptor (ER), progesterone receptor (PR) and the overexpression or gene amplification of human epidermal growth factor receptor 2 (HER2). The expression profiles of these markers have led to three main clinical breast cancer subgroups for which treatment regimes are based; ER + /HER2-, HER2 + or triple-negative breast cancer (TNBC) which lack ER, PR and HER2 expression^3,4^. The seminal studies using gene expression profiling, have further subdivided breast cancers into molecular and transcriptomic subtypes, which are of prognostic and predictive importance^5–9^. Together, these studies highlight the innate inter-TH amongst breast cancer patients.

Intra-TH escalates the complexity of the disease in many aspects including our understanding of cancer development, progression, metastasis, therapy response and acquired drug resistance. Two non-mutually exclusive models of intra-TH have been proposed, the cancer stem cell model and the clonal evolution model^10^, which are extensively reviewed elsewhere^2,11–13^. Despite the development of next-generation sequencing having enabled in depth analysis of intra-TH and tumour evolution, these two factors contribute to systemic treatment failure by initiating phenotypic diversity enabling drug resistance to emerge.

In this review, we summarise the importance of next-generation sequencing in identifying intra-TH in breast cancer at a genetic level, identify the clinical impacts and challenges of intra-TH to patients’ treatment outcomes and how genetic intra-TH can be assessed by single-cell approaches. Furthermore, we explore the non-genetic forms of intra-TH during breast cancer evolution, drug resistance and more importantly how single-cell approaches in conjunction with appropriate preclinical models can be used to untangle the complexity of the disease.

### Genetic intra-TH in breast cancer

In the past decade, through the development and implementation of next-generation sequencing, numerous studies have reported the existence of intra-TH in breast cancer at the genetic level. This has been attributed to chromosomal or genomic alterations affecting biological processes and functions^3^. For instance, through large-scale genome analysis of primary human breast cancers, the complex mutational landscape of breast cancer has been mapped, highlighting the existence of clonal tumour subpopulations in breast cancer^14–22^. These studies in general have highlighted that a large majority of mutations detected in primary breast cancers are subclonal^17^ and not necessarily evenly distributed spatially among individual tumours highlighting the need for multiple sampling^21^. In primary untreated breast cancers, multi-region sequencing has highlighted that the extent of subclonal diversification varies considerably among individual tumours with no strict temporal order being evident, and genomic alterations such as point mutations and rearrangements affect the most common breast cancer genes, including *PIK3CA*, *TP53*, *PTEN*, *BRCA2* and *MYC*, occurring early in some tumours and late in others^21^. This is in contrast with other tumour types that show a specific temporal order of mutations^23–26^. This may be due to the fact breast cancers inherently are thought primarily to be driven by an array of copy number driver alterations, with most somatic mutations occurring later in tumour development^19^ or may reflect a situation wherein some tumours that grow to a certain size, clonal sweeps occur in which an especially fit subclone replaces the majority of others in the tumour^21^. As the different subtypes of breast cancer are thought to arise from different cells-of-origin, this may additionally explain the differences in heterogeneity observed in the different subtypes of breast cancer^5^.

### Tracking subclonal mutations in patients undergoing therapy

**Fig. 1:**
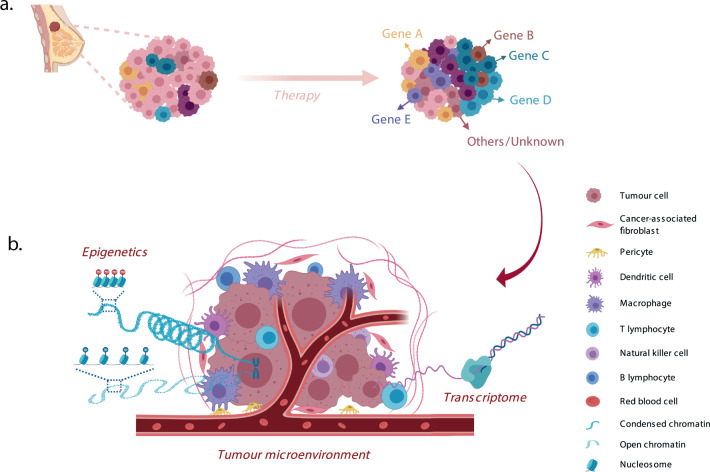
Pre-existing and acquired mechanisms of resistance to therapy in a model of clonal selection. **a** Tumours consist of genetically and phenotypically diverse clones (represented in different colours). Selective pressure such as therapy will eliminate a percentage of sensitive clones leaving behind some pre-existing resistant clones with particular genomic aberrations. These clones are able to survive and expand leading to drug resistance mediated through the selection of pre-exitsing clones (represented as gene A–D). New genomic aberrations may also arise due to the selective pressures of therapy leading to acquired drug resistance (represented as gene E). Remaining clones that are resistant to therapy may also be due to additional unknown resistance mechanisms and they may be pre-existing and/or acquired. **b** Pre-existing and/or acquired drug resistance may be due to transcriptional and epigenetic mechanisms affecting gene expression or the communication of tumour cells with the microenvironment. This figure was created with BioRender.com and is adapted in part from the template 'tumour microenvironment 2'.

Subclonal changes can additionally occur upon the selective pressure of therapy (Fig. 1a)^19,21,27–30^. Drug resistance can be pre-existing (where therapy does not impact subclone frequency), selected (arising to the selection of rare pre-existing subclones that are able to expand), or acquired (where new genomic/transcriptomic/epigenetic aberrations are introduced contributing to the drug-resistant phenotype). Relapsed or metastatic breast cancers have been found to largely share the vast majority of their genomic alterations with the corresponding primary disease indicating pre-existing resistant clones, however many metastatic tumours also exhibit additional mutations that were not previously detected or are subclonal in the primary disease^21,31–37^. As an example, *ESR1* activating mutations are rarely present in primary ER + breast cancer, even in those 15–20% patients that show intrinsic resistance to hormonal therapies^22,38,39^, whereas they are highly enriched in ER + metastatic tumour samples from patients with acquired endocrine resistance (30–40% of ER + patients) and in particular, are a known mechanism of resistance to aromatase inhibitors (AI)^40–42^. Recent large studies cataloguing the mutational repertoire of primary and relapsed metastatic breast cancers from patients have highlighted that multiple mechanisms of endocrine therapy resistance exist in ER + BC cancers^43^ including evidence that pre-existing low-frequency mutations can be responsible for endocrine resistance^44,45^.

Genetic mechanisms of resistance to chemotherapy are less well-documented in TNBC, and in general subclonal alterations are not enriched upon chemotherapy treatment^27,46^ highlighting that while a proportion of patient’s tumours undergo clonal selection, others maintain a similar genomic architecture after chemotherapy^46,47^. It is worth mentioning that poly(ADP)-ribose polymerase (PARP1/2) inhibitor resistance is also observed in patients with homologous recombination defects including TNBC^48–52^. Although PARPi-based therapy is standard of care for patients with germline *BRCA1/2* mutations including breast and ovarian cancer, resistance has been observed in these patients, where reversion mutations of *BRCA1* or *BRCA2* are a well-documented resistance mechanism^53,54^ and can be assessed through profiling of circulating free DNA (cfDNA)^55^. Although a plethora of potential resistance mechanisms to PARPi have been identified through high throughput functional genomic screens, many of these have yet to be validated in patients relapsing on PARPi treatment^48–52,56,57^. Overall, whether these reversion mutations are spontaneous, pre-existing and selected for under PARP1/2 inhibition, or induced by other therapeutic agents such as platinums previously administered to patients is still to be determined.

As well as bulk profiling of tumour biopsies, assessment of circulating tumour (ct) or cfDNA of cancer patients is a useful evolutionary methodology to track the evolution of intra-TH of cancer, the presence of treatment-resistant clones and to predict response and resistance. These tools have been shown to be useful in both metastatic patients^58^ and in early breast cancer^59^ to monitor response to therapy and are predictive of relapse^42,60,61^. Importantly, ctDNA analysis can provide important genomic information that cannot be traced by a single biopsy at a specific cancer site and are becoming increasingly used in clinical studies as a non-invasive way of assessing the emergence of progressive disease and to direct therapy^62–64^. Although further research is necessary to determine whether ctDNA can fully represent tumour genomic profiles at any given time, recent data from a seminal clinical trial, plasmaMATCH, has assessed the validity and utility of ctDNA for targeted therapies without previous tissue testing in advance breast cancer patients^65^. Novel methylation profiling methods (so-called ‘molecular clocks’) can allow tracking of evolutionary changes of single CpG sites to track clonal haplotypes in ctDNA (‘epimutations’ that accumulate due to methyltransferase errors at cell division and occur 1000 to 10,000 times more frequently than DNA point mutations) at single-molecule resolution without a priori knowledge of the molecular make-up of the disease^66^. Implementation of such methodologies will likely overcome the limitations in terms of cost and prior knowledge of a patients’ tumour mutational repertoire for successful longitudinal patient tracking.

### Assessment of genetic intra-TH using single-cell approaches

Single-cell approaches to assess intra-TH have traditionally used florescence in situ hybridisation to measure copy number using probes against frequently amplified loci in breast cancer. Using diversity measures one study seeking to address whether copy number alterations drive neoadjuvant chemotherapy (NAC) resistance revealed that tumours that respond to NAC have a low diversity of copy number alterations before therapy, whereas those that had a poor response showed a higher diversity^67^. However, these patterns were unchanged between pre- and post-treatment biopsies, suggesting perhaps resistance to NAC is not driven by particular genomic alterations. Interestingly, this study did identify intra-TH based on the expression of cancer stem cell markers such as CD44 and CD24, that correlated with NAC response, suggesting phenotypic evolution plays a role in chemotherapy resistance^67^. Such studies have also highlighted that the spatial pattern of *PIK3CA* mutations together with *HER2* amplified cells play an important role in chemotherapy resistance^68^.

Recent advances in single-cell sequencing technologies now allow the assessment of intra-TH of tumours, as well as the non-genetic changes that may occur upon metastatic disease progression and upon selective pressures such as chemotherapy and targeted therapy in breast cancer. Single-cell sequencing overcomes the need for computational deconvolution of the theoretical contribution of individual clones but instead can profile the DNA, RNA, methylation and chromatin accessibility of each individual cell^30,69–71^.

Selective pressures such as targeted therapy can also alter the transcriptome of cancer cells, leading to drug resistance and patient relapse. In the first seminal single-cell sequencing study from Navin and colleagues, single-cell profiling was used to assess copy number heterogeneity in two breast cancer patients^72^. This study revealed that polygenomic tumours likely represent sequential clonal expansions and that seeding of metastasis from a monogenomic tumour and its liver metastasis indicated that a single clonal expansion formed the primary tumour and seeded the metastasis. Interestingly, the authors found that an unexpectedly abundant subpopulation of genetically diverse ‘pseudodiploid’ cells that did not travel to the metastatic site, suggesting that tumours grow by punctuated clonal expansions with few persistent intermediates in contrast to more gradual models of evolution^72^. A follow-up study looking at mutations at the single-cell level identified that copy number alterations occurred early in tumour evolution and remained highly stable suggesting clonal expansion, whereas, in contrast, point mutations evolved gradually, generating extensive clonal diversity, with many mutations seen in less than 10% of the tumour cells^30^. Application of this technology to patients with ductal carcinoma in situ and concurrent invasive disease using topographical single-nucleus sequencing has revealed that the majority of genomic alterations developed in the ducts prior to the invasion, supporting a multiclonal invasion model, whereby more than one clone escapes to establish the invasive disease^73^. A similar study using single-cell profiling of formalin-fixed paraffin-embedded clinical tumour samples to assess copy number of the transition from ductal carcinoma in situ to invasive disease also found extensive intra-TH in pre-invasive lesions with evolution to invasive disease occurring via multiple mechanisms^74^. Together, these studies suggest genomic diversification of copy number alterations occur early during tumorigenesis due to clonal expansions and subclonal mutations occur later on, generating substantial mutational heterogeneity.

### Contribution of non-genetic forms of heterogeneity

Although the majority of the studies so far have focussed on assessing genetic alterations (mutations and copy number) to evaluate intra-TH, as discussed above, there is emerging evidence that non-genetic factors also play a major contributing role. Numerous studies have suggested the existence of differential transcriptomic make-up between primary tumour and the corresponding metastatic lesions^75–78^. Drug resistance can be through the selection of pre-existing alterations and/or acquired due to transcriptional and epigenetic mechanisms such as histone modifications and DNA methylation affecting gene expression or communication of tumour cells with the microenvironment (Fig. 1b)^15,46,47,79–85^. This has been addressed in a number of studies evaluating the single-cell transcriptomic make-up of the normal epithelium and of primary breast cancers.

Transcriptomic analyses from primary and metastatic cancers at both bulk and single-cell level has further highlighted the complexity of tumour evolution. Several reports have shown that metastatic breast cancer lesions show a higher transcriptomic heterogeneity than the corresponding primary disease^75–78,86^. Single-cell sequencing of circulating tumour cells (CTC’s) of patients with metastatic breast cancer versus patients with primary breast cancer, highlighted increased transcriptomic heterogeneity in those patients^76^. Of note transcriptional signatures associated with epithelial to mesenchymal transition were enriched in CTC’s derived from patients with metastatic disease^76^. Importantly, a single-cell study of patient-derived TNBC primary tumours in vivo and their corresponding lung and lymph-node micro-metastases exhibited a distinct transcriptome at a single-cell level. Interestingly, the mitochondrial oxidative phosphorylation (OXPHOS) pathway was upregulated in micro-metastases^75^. Notably, pharmacological inhibition of OXPHOS attenuated lung metastasis in vivo, highlighting the importance of this pathway in a metastatic spread as well as its potential in metastatic prevention in breast cancer patients^75^. These findings are in line with bulk RNA sequencing of primary tumours and their matched metastases of TNBC patients, highlighting consistent gene expression differences including enrichment of hypoxia, cellular metabolism, and fatty acid β oxidation pathways in the metastases, and were independent of the metastatic site^86^. Collectively, the above studies indicate the importance of transcriptomic heterogeneity in metastatic breast cancer as well as its potential role in the aggressiveness of this disease.

In a seminal study from Navin and colleagues, single-cell DNA and RNA sequencing was used to analyse the contribution of genetic and non-genetic heterogeneity in TNBC patients receiving NAC and treatment with the anti-VEGF therapy (bevacizumab)^47^. Although this study only looked longitudinally in a small number of tumours, some patients harboured enrichment of genomic alterations in residual disease post-NAC, however, overall, there was no evidence that specific genetic differences contributed to chemotherapy resistance. Single-cell RNA sequencing of a subset of these tumour samples, however, highlighted that the transcriptomic profiles of pre-treatment and post-treatment samples were distinct, suggestive of a model of therapy resistance in which selective and acquired modes of evolution are responsible for the resistant population, in agreement with previous in situ based studies^67^.

A recent study performing single-nucleus RNA sequencing of six therapy naïve primary TNBC fresh tumours highlighted that in the absence of chemotherapy therapeutic pressure, TNBC’s show extensive intra-TH at the transcriptomic level^87^. This appeared to be driven by copy number alterations within the cells, suggestive that at least in the primary disease setting, the genotype can influence transcriptomic heterogeneity of individual subpopulations. Perhaps most interesting from this study was the identification of distinct subclonal populations of malignant cells that were shared between the different patients characterised by multiple signatures of treatment resistance and metastasis, including glycosphingolipid metabolism and associated innate immunity pathways. Moreover, these signatures identified from the subclonal cellular populations were able to predict long-term prognosis in independent cohorts of primary TNBC from bulk RNA-profiling. This suggests that minority populations of cells have already acquired the transcriptomic features to allow them to become metastatic. Moreover, if this signal can be elucidated from bulk tissue once identified, this heralds a paradigm shift for identification and specific targeting of these subpopulations a priori to circumvent metastatic spread.

A number of recent studies have attempted to address whether drug resistance including endocrine therapy or chemotherapy in breast cancer is selected for and/or acquired. Upon endocrine therapy in ER + breast cancer cell line, MCF7, a rare subpopulation of pre-adapted cells with high plasticity have been found to have district transcriptomic signatures (upregulation of genes involved in p53 signalling pathway, epithelial-mesenchymal transition (EMT), hypoxia and cell polarity) with features of dormancy as well as mixed epithelial and mesenchymal characteristics. Interestingly, single-cell sequencing highlighted that these pre-adapted cells that display features of dormancy and mixed epithelial and mesenchymal traits are able to survive upon short-term endocrine therapy, however, these pathways are not seen in fully resistant cells, suggesting that the cells undergo further transcriptomic reprogramming to become fully resistant to endocrine therapy and to generate subsequent metastasis^79^. Additionally, single-cell sequencing of ER + cell lines has identified genetically and transcriptionally distinct subpopulations including rare populations of MCF7 cells highly expressing an apoptosis-related signature, positively correlated with a pre-adaptation signature to oestrogen deprivation^88^. These results suggest a multi-step mechanism of drug resistance to endocrine therapy where both genetic and nongenetic alterations contribute to this phenotype. In a similar study, authors used molecular barcoding coupled with single-cell transcriptomic sequencing to track the ER + cell line, MCF7, upon long-term endocrine therapy in vitro. Their findings suggested that the majority of fulvestrant or tamoxifen resistance clones were pre-existing and highly selected during treatment^80^.

Single-cell RNA profiling of CTC’s from liquid biopsies have further revealed the extent of intra-TH, with rare CTC’s displaying divergent gene expression signatures of EMT, cancer stemness, DNA repair, kinase signalling and immune-tumour cross-talk^89^. Moreover, single-cell profiling of CTC clusters identified that these clusters are oligoclonal, and although rare have up to a 50-fold increase in metastatic potential and are characterised by high expression of the cell junction component plakoglobin, suggesting this could be a useful biomarker to detect cells with high metastatic potential in the primary tumour^90^. Profiling of CTC’s from metastatic breast cancer patients has also highlighted the intra-TH of *ESR1* mutations in patients with endocrine resistance suggesting independent mechanisms of resistance to these agents, and highlighting the clinical applicability of single-cell approaches for detecting genomic alterations mediating targeted therapy resistance^91^.

### The role of epigenetic heterogeneity in therapy resistance

It is now well established that epigenetic mechanisms play a vital role in driving phenotypic heterogeneity and evolution and contribute to treatment resistance in breast cancer patients. In endocrine resistance in ER + MCF7 breast cancer cells, for example, drug-induced epigenetic states have been shown to involve large topologically associating domains and the activation of super-enhancers. In particular, MCF7 cells resistant to AI have been shown to upregulate cholesterol biosynthesis through stable epigenetic activation, which leads to constitutive ER-activation in AI-resistant cells^82^. More recently, enhancer mapping of H3K27 acetylation states in 47 matched primary and metastatic endocrine-resistant tumour biopsies has allowed the inference of tumour evolution and highlighted that endocrine therapies select for phenotypic clones under-represented at diagnosis^92^. These studies suggest phenotypic heterogeneity governed by inheritable cell-type-specific transcription is maintained through the activation of epigenetically defined regulatory regions including promoters and enhancers^92^.

Evidence also suggests epigenetic intra-TH governs resistance to novel therapeutic agents such as Bromodomain and Extra-Terminal motif inhibitors^93^. Indeed, alterations in epigenetic mechanisms through the induction of several transcriptional states, contribute to intra-TH and thus to the aggressiveness of the disease and drug resistance^94^. In a recent pivotal study by Hinohara and colleagues, the epigenetic enzyme, KDM5B, was found to be a regulator of transcriptomic heterogeneity in a panel of ER + breast cancer cell lines, promoting resistance to endocrine therapy. Its high expression in ER + breast cancer cell lines was found to be associated with higher transcriptomic heterogeneity^80^ and was reversed upon treatment with KDM5 small molecule inhibitors, providing proof of concept that modulating the activity of epigenetic enzymes, can lead to improved responses to the standard of care treatment. Interestingly in cellular models, resistance to KDM5 inhibitors was acquired rather than selected for as in endocrine therapy resistance. Additional studies have also identified epigenetic reprogramming of the transcriptional repressive mark, H3K27me3, in cell populations resistant to chemotherapy in TNBC and to tamoxifen in ER + breast cancer patient-derived xenografts (PDXs)^83^.

### Single-cell approaches to dissect the contribution of the tumour microenvironment

Increasing evidence suggests that epigenetic and subsequent transcriptomic alterations in cells within the tumour microenvironment (e.g. stromal, immune cells and non-cellular components that make up the extra-cellular matrix) contribute to tumour initiation, progression and metastasis mediated through paracrine signalling with the tumour cells^95,96^. Gene expression signatures derived from the tumour stroma can be predictive of patients’ clinical outcome^97^. Thus, there is increasing interest in addressing the contribution of the microenvironment in terms of tumour development, progression, drug resistance as well as its potential as a prognostic factor and a therapeutic target. The use of single-cell RNA sequencing has enabled deeper insights into intra-TH as well as the contribution of tumour microenvironment which are not detectable by other methods^98–100^. Recently, the immune atlas in breast cancer has been mapped. Using unbiased single-cell RNA sequencing analysis, the immune cells from eight primary breast cancers patients, as well as their matched normal breast tissue, blood and lymph nodes were profiled in order to investigate the role of immune cells in tumour microenvironment and its contribution to cancer progression and immunotherapy response^98^. The authors identified significantly increased phenotypic expansion of intra-tumoral immune cells in comparison to normal breast tissue. Similarly, the tumour microenvironment of primary TNBC tumours has been profiled by high throughput single-cell RNA sequencing^100^. The authors identified two cancer-associated fibroblast and two perivascular-like subpopulations, where each population clustered into different states. These signatures were found to be strongly associated with cytotoxic T-cell dysfunction, leading to immune evasion^100^.

It is becoming clear that the spatial organisation of tumour microenvironments plays an important role in therapy response and patient outcome in breast cancer^101,102^. Tissue micro-dissection paired with gene expression analysis has provided valuable insights into tumour–microenvironment relationships. For example, it was found that in TNBC T-cell infiltration within a tumour mass, but not peripheral localisation of T cells, was associated with good patient outcome^103^. Digital pathology coupled with computational assessment allows a unique insight into how the tumours are organised and is an exciting emerging field. For example, the spatial heterogeneity of immune cells, stromal cells and epithelial cancer cells assessed by haematoxylin and eosin routine histopathology sections has been shown to be associated with a poor response to standard therapies in both ER + and ER- primary breast cancers, suggesting that having a mixture of these cells within the tumour microenvironment allows adaptation as the cancer evolves^102^. Moreover, increased immune cell spatial clustering has been shown to predict both early (0–5 years) and late (5–10 years) recurrence in ER + breast cancer patients after endocrine therapy, whereas total immune cell abundance showed no such an association^101^. A recent study of 20 primary breast cancers subjected to single-cell RNA coupled with epitope sequencing (CITE-seq), has revealed new PD-L1/PD-L2 + macrophages associated with clinical outcome^100^. Using spatial transcriptomic sequencing on the corresponding tissue sections of these tumours further revealed that stroma-immune niches were spatially organised. Moreover, single-cell signatures were able to stratify large breast cancer cohorts at the bulk RNA level into nine “ecotype” clusters associated with unique clinical outcomes which were distinct from the classical intrinsic molecular subtypes of breast cancer^100^. Recent technological advances in proteomics have additionally allowed assessment of the epithelial and stromal cell make up of primary breast cancers whilst maintaining the spatial architecture. Using imaging cytometry time of flight (CyTOF) to measure multiple proteins in a single experiment now allows unprecedented assessment of the tumour microenvironment. These studies have revealed the extensive tumour and immune ecosystems in primary breast cancers^104,105^ and highlighted associations between genomic alterations such as *TP53* mutations and distinct epithelial and stromal phenotypes, that hypoxic environments may induce immune tolerance, and identified that single-cell pathology subgroups have distinct clinical outcomes^104,105^. Further assessment of these during tumour evolution will be important going forward to validate these as biomarkers of disease progression.

### Potential clinical impacts of intra-TH in breast cancer

Whilst genetic intra-TH has been documented to be associated with poor patient outcomes in tumour types such as leukaemia and lung cancer^106,107^, its prognostic association in breast cancer is less clear. Multi-regional sequencing analysis of 75 TNBC primary tumours suggested genomic intra-TH of copy number status in several driver oncogenes is correlated with metastasis^108^. In the context of evolution from primary to metastatic disease, high depth bulk profiling of primary and matched metastatic tumours from the same patient have revealed that most distant metastases acquire genomic driver mutations not seen in the primary tumour, drawing from a wider repertoire of cancer genes than early drivers, including a number of clinically actionable mutations inactivating SWI-SNF and JAK2-STAT3 pathways^36^. In the context of treatment response, analyses of relatively small cohorts using multi-regional profiling have revealed contrasting results associated with therapy responses, with one study suggesting correlation^67^ and another study finding lack thereof^21^ between the extent of genomic intra-TH with chemotherapy response. In a study assessing the mutational repertoire in primary HER2 amplified breast cancers with heterogeneous HER2 protein expression, the clinical impact of intra-TH is perhaps clearer. Heterogeneous HER2 amplification is seen in around 5% of HER2 amplified breast cancers. Through bulk dissection and genomic profiling of HER2-amplified and non-HER2 amplified components of clinically classified HER2-positive breast cancers, a recent study identified that the HER2-negative component of these tumours harboured additional driver mutations, including one case with an activating HER2 mutation, highlighting convergent evolution in this patient. Overall, however, the HER2-negative component would not be predicted to respond to anti-HER2 therapies, this highlights the need for close monitoring of these patients undergoing HER2 targeted therapeutic interventions^109^. Further assessment of the role of phenotypic heterogeneity driving the prognosis of breast cancers from single-cell epigenomic and transcriptomic studies is starting to reveal that transcriptomic features are seen in minority subpopulations and novel immune subpopulations in primary disease correlates with patient outcome^87,100^. There is a major need in the field to conduct large-scale survival analysis with genome-wide indicators of intra-TH to thoroughly address the question of whether primary tumour intra-TH is truly associated with patient outcomes in breast cancer.

### Modelling intra-TH and subclonal evolution

The observed intra-TH over the past years in breast cancer has raised the need for the generation and use of relevant preclinical models mirroring the full spectrum of the disease. Although established cancer cell lines have served as a fundamental in vitro model in breast cancer research over the past decades, they do not fully recapitulate all aspects of breast cancer including the natural structure of tumours, altered cell morphology, microenvironment, molecular and genomic resemblance to breast cancer tumours^110,111^. The generation and use of PDXs, PDX derived organoids (PDXOs)^110–114^, and patient-derived organoids (PDOs)^115^ from surgical or biopsy patients’ samples have proven to be a very powerful, preclinical in vitro and in vivo tool to assess drug response and resistance, modelling metastasis, partially elucidate tumour microenvironment and for biomarker development as they more accurately represent the patients’ tumours. Importantly, PDXs^33,78,116–118^ and PDXOs^115^ have both been shown to preserve much of the intra-TH found in originating patients’ biopsies, making them attractive models with which to investigate intra-TH dynamics in the laboratory. PDXOs and PDOs are currently attractive models with which to readily probe the activities of hundreds of experimental compounds in patient-derived breast tumour cells^115,119^. The primary limitation of PDX models is the lack of a fully intact immune system. Thus, future studies implementing pharmacologic and genetic screening technologies in models with ‘humanised’ immune systems will be of great value to the field.

Many scientists now use PDXs and PDOs as preclinical models to address intra-TH and evolution in cancer (see ref. ^120^ for an extensive review). One methodology in achieving this is lineage tracing by using highly complex barcode libraries, enabling the tracking of individual cells or subclones during both the metastatic cascade and through therapy resistance, using next-generation sequencing as a readout^121^. These technologies label up to millions of cells with unique molecular barcode identifiers that allow transmission during cell division and hence single-cell tracing over time. This approach allows the investigation of the presence of pre-existing resistant clones in the tumour mass, molecular changes that can occur due to therapy and resistance, or therapeutic approaches to be taken into consideration to successfully target these pre-existing resistant clones through single-cell DNA or RNA sequencing^46,79,88,94,122^. This method in particular has been used to study clonal dynamics and therapy response in primary TNBC^46,78,123^. In these elegant studies, patient-derived models of primary TNBC were used and cells were barcoded with a complex lentiviral library. Residual tumours after chemotherapy exposure were found to maintain their barcode complexity rather than show a pattern of enrichment, suggestive of cellular plasticity, rather than pre-existing genomic alterations that emerged. OXPHOS was one of the most significantly upregulated pathways in residual tumours and treatment with an inhibitor of the mitochondrial electron transport chain complex I delayed the regrowth of chemotherapy-treated residual tumours in vivo. Interestingly, residual PDX tumours had differentially activated epigenetic regulators including HDAC7, HDAC10 and SIRT7, suggesting that these epigenetic regulators can act as promising candidates for TNBC tumours with residual disease in combination with metabolic inhibitors^46^. In vivo barcoding in primary untreated TNBC PDX models has also allowed investigation of the subclonal evolution of metastatic spread. These studies harness the utility of the fact that many TNBC PDX’s spontaneously metastasise to distant organs in the absence of selective pressure such as chemotherapy. Using the same barcoding approach, Echeverria and colleagues showed that metastatic spread occurs through a selective bottleneck revealing an enrichment of shared clonal lineages across multiple metastatic sites, indicative of polyclonal seeding. Interestingly, rather than only a few clones leading to seeding at distant sites, the authors also observed extremely rare populations of cells that were able to seed and were maintained at low levels at these metastatic sites^78^. Similarly, studies using barcoded syngeneic 4T1 mammary tumour derived cells have demonstrated a bottleneck in metastatic seeding from the primary tumour in CTC’s analysed in the blood and cells at distant sites. Interestingly in this model, these lineages were largely non-overlapping suggestive of independent subclones colonising distant sites and showed tropism for entering the lymphatic or vasculature system. Vascular tropism in particular was correlated with increased expression of Serpine2 and Slpi, which were necessary and sufficient to programme these cells for vascular mimicry^124^. In addition, molecular barcoding has been used coupled with CRISPR technologies to trace a cell phenotype immediately after the introduction of DNA mutations in cancer^125,126^.

Given the polyclonal nature of metastatic spread and the fact CTC clusters have been shown to have a substantial increase in metastatic seeding^90^, this highlights the possibility that subclones interact with one another to mediate therapy resistance and spread. This is in where one subclone may maintain the fitness advantage of others through distinct paracrine contributions by maintaining the fitness of the whole or subpopulations resulting in a clonally diverse tumour phenotype. This concept has been evaluated using a Trp53-null mouse model of basal-like breast cancer, where a unique subpopulation of tumour cells expressing mesenchymal-like markers was identified to cross-talk with tumour initiating cells promoting tumorigenicity^127^. Cleary et al. show that, in some *Wnt1* driven mammary tumours, neither luminal nor basal cancer cells alone could form a new tumour when injected into the mammary fat pads of immunocompromised mice, whereas mixtures of the two cell populations induced biclonal tumour formation with high efficiency, suggestive of cooperativity between the two cellular populations^128^. More recently, in vivo assessment of isolated secreted factor-overexpressing subclones of the TNBC cell line, MDA-MB-468, revealed non-cell-autonomous maintenance of intra-TH^129^ in which minor subclones expressing IL11 and FIGF (VEGFD) cooperate to promote metastatic progression and generate polyclonal metastases composed of a driver and neutral subclones. This cooperation was indirect and mediated through a specific neutrophil population that was stimulated by the IL11-expressing minor subclone^130^. Together, these studies demonstrate that non-cell-autonomous cooperative interactions exist between different cellular populations within tumours and highlight potential intervention strategies to circumvent these. In the context of NAC resistance in TNBC in particular, subclonal cooperativity may explain the reason for the lack of recurrent molecular alterations identified in NAC-resistant tumours.

**Fig. 2:**
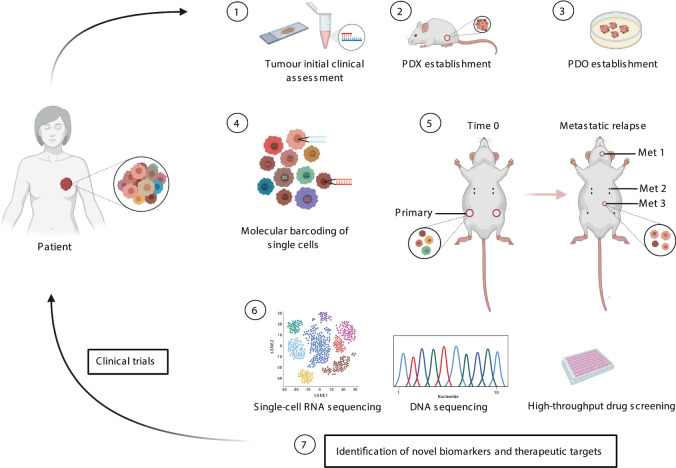
Models tackling intra-TH in breast cancer. Once the primary tumour is removed from a patient, the tumour sample is used for (1) initial clinical assessment and characterisation, followed by (2) PDX (patient material grown in mice) and (3) PDO (patient material directly grown ex vivo) establishment for research purposes and compared to the primary tumour sample. The remaining tumour is (4) dissociated to single cells, barcoded and (5) injected into the mammary glands of mice. Primary and matched metastatic tumours are removed from mice and subjected to (6) single-cell RNA sequencing, DNA sequencing and high throughput drug screenings thus (7) identifying the genetic, non-genetic make-up and evolution of these tumours at a single-cell level as well as identifying novel therapeutic strategies and biomarkers. This is then linked back into the analysis of patient samples in clinical trials. This figure was created with BioRender.com.

Taken together, these studies highlight the polyclonal and complex nature of metastatic spread in breast cancer. Further assessment of these in humanised patient-derived models to encompass competent immune systems will be necessary to further elucidate mechanisms of metastatic spread and resistance to particular therapies. One can envision a strategy whereby this could be intersected with an extensive characterisation of clinical tumour sampling at single-cell resolution in matched models to fully assess the intra-TH at a genetic and genomic level in an individual patient. Only then, we will be in a better place to tackle the complexity of intra-TH and evolution in cancer (Fig. 2).

## Conclusion

Intra-TH remains a challenge in terms of appropriate cancer therapeutic strategies. Current biomarker analysis does not fully represent the complexity of TH; the existence of rare subclones or accurately predicting treatment outcomes of cancer patients. Deconvoluting the genomic, epigenomic and functional heterogeneity in breast cancer can improve our understanding of the drivers of the disease, mechanisms of resistance and metastasis, identify more accurate biomarkers and novel therapeutic strategies. The use of advanced methodologies such as molecular barcoding, single-cell RNA profiling together with mathematic modelling have proven to be of high importance in terms of addressing these issues. However, further efforts are necessary in order to comprehensively determine and control intra-TH in breast cancer. Furthermore, the development of novel cfDNA molecular tracking such as the use of ‘molecular epigenetic clocks’, can provide unbiased tools for longitudinal patient monitoring in the clinic.

The continuous transcriptional and epigenetic contribution during cancer progression or upon selective pressures have implications on the gene expression of tumour cells, making the tumour more heterogeneous and thus difficult to treat, leading to drug resistance and metastasis. Although the development of advanced methodologies such as single-cell RNA sequencing and single-cell tracing molecular barcoding methodologies in relevant preclinical models have significantly improved our understanding of TH and evolution, further studies in immune-competent models are necessary in order to map the genomic and epigenetic landscape of these rare drug-resistant clones. Moreover, additional studies are needed to fully appreciate the potential inter-clonal cooperativity mediated by both non-tumour cells and between different tumour subclones. This will then allow insights into the identification of novel drugs that can disrupt these interactions and shift these clones from a drug-resistant state to a drug-responsive state. Additionally, the transcriptomic profiles from the studies described above can be applied as tools to predict which patients will respond to specific therapies, provide prognostic signatures for patients’ outcomes besides the histopathological techniques already in use, as well as promising candidates for new therapeutic strategies in breast cancer. These could be used as novel combinatorial approaches together or using intermittent scheduling approaches with existing therapies including immuno-modulatory agents targeting the tumour microenvironment.
